# Removal of multifocal neuroendocrine lung tumours with a LIMAX^® ^120 Nd:YAG laser: case report

**DOI:** 10.1186/1749-8090-10-S1-A23

**Published:** 2015-12-16

**Authors:** Andreas Kirschbaum, Peter Rexin, Anja Rinke, Detlef K Bartsch

**Affiliations:** 1 Department of Visceral-, Thoracic- and Vascular Surgery, University Hospital Marburg, D-35033 Marburg, Germany; 2 2 Institute of Pathology, University Hospital Marburg, D-35033, Marburg, Germany; 3 Gastroenterology, University Hospital Marburg, D-35033, Marburg, Germany

## Background/Introduction

Multifocal neuroendocrine lung tumours are rare. When they are nonetheless diagnosed there is uncertainty as to how treatment should proceed. We present such a case. Our decision was to surgically remove all the lung foci visible on thoracic computer tomography.

## Aims/Objectives

The patient was a 69-year-old woman. Computer tomography of the thorax carried out after a road traffic accident revealed bilateral lung foci measuring up to 1.5 cm. The patient showed no lung symptoms.

## Method

A transbronchial biopsy was not able to clarify the cause of the foci. Two of the lung foci were removed by non-anatomical resection using video thoracoscopy. Histological examination of the material surprisingly revealed two typical carcinoid tumours (Ki67 index < 1%). As all the remaining foci were considered to be resectable the interdisciplinary tumour board recommended resection of all lesions. We performed open bilateral resection on a total of 14 foci (six in the right lung and eight in the left). Each of the lung foci was removed non-anatomically in sane with the LIMAX^® ^120 (Gebrüder Martin & CoKG, Tuttlingen, Germany) diode-pumped Nd:YAG laser. Radical mediastinal lymphadenectomy was also carried out bilaterally. None of the X removed lymph nodes showed metastatic foci. The operations were carried out 4 weeks apart. Postoperative complications did not occur.

## Results

Three years later, imaging revealed neither local recurrence nor new lung foci.

## Discussion/Conclusion

Multifocal neuroendocrine lung tumours are often diagnosed coincidentally. If they appear to be resectable, the goal should be non-anatomical resection of all foci. The Nd:YAG laser LIMAX^® ^120 is special suitable for this, even with a large number of tumours. In the most favourable case a lasting cure can be achieved.

## Consent

Written informed consent was obtained from the patient for publication of this abstract and any accompanying images. A copy of the written consent is available for review by the Editor of this journal.

